# Successful Xenogeneic Transplantation in Embryos: Induction of Tolerance by Extrathymic Chick Tissue Grafted into Quail

**DOI:** 10.1155/1991/57259

**Published:** 1991

**Authors:** Claude Martin, Hiroko Ohki-Hamazaki, Catherine Corbel, Monique Coltey, Nicole M. Le Douarin

**Affiliations:** 1 lnstitut d'Embryologie cellulaire et moléculaire du CNRS et du Collège de France, 49bis Avenue de la Belle-Gabrielle Nogent-sur-Marne Cédex 94736 France; 2 School of Human Sciences Waseda University 2-579-15 Mikajima Tokotozawa Saitama 359 Japan

## Abstract

In previous experiments, we have demonstrated that limb buds engrafted during embryonic life at E4, between MHC-mismatched chick embryos, are not only tolerated after birth, but induce in the recipient a state of split tolerance toward cells expressing the donor MHC haplotype: donor's skin grafts are permanently tolerated while a proliferative response of host's T cells is generated in MLR by donor–type blood cells. If the same experiment is performed, using quail embryo as a donor and chick as a recipient, acute rejection of the quail limb starts during the first two weeks after birth, thus suggesting that the peripheral type of tolerance induced in these experiments can be obtained only in allogeneic but not in xenogeneic combinations.

We report here the unexpected result that when a chick limb bud is grafted into a quail at E4, it is tolerated and, like allogeneic grafts in chickens, induces adult skin-graft tolerance without modifying the MLR response. Similar results were obtained with grafts from another closely related species of bird, the guinea fowl from the *Phasianidae* family. In contrast, xenogeneic combinations involving more distant species (chick and quail as recipients and duck, an *Anatidae*, as donor) resulted in strong and early rejection from both recipients. As a whole, quails exhibit a greater ability than the chick to become tolerant to antigens presented peripherally from early developmental stages. In adult quails, however, skin grafts performed in either direction (i.e., quail to chick or the reverse) are rejected according to a similar temporal pattern. Moreover, lymphocytes of both species are able to respond equally well to quail or chick IL-2. Several hypotheses are envisaged to account for these observations. It seems likely that this type of tolerance is directly related to antigenic load because the load in chick to quail wing chimeras is larger than that in quail to chick chimeras. This view is supported by the protracted delay in graft rejection observed when two quail wing buds instead of one are grafted into chickens.


*Developmental Immunology*, 1991, Vol. 1, pp. 265–277
Reprints available directly from the publisher
Photocopying permitted by license only

© 1991 Harwood Academic Publishers GmbH
Printed in the United Kingdom

### Successful Xenogeneic Transplantation in Embryos: Induction of Tolerance by Extrathymic Chick Tissue Grafted into Quail

CLAUDE MARTIN,† HIROKO OHKI-HAMAZAKI,‡ CATHERINE CORBEL,† MONIQUE COLTEY,† and NICOLE M. LE DOUARIN*†

*†Institut d'Embryologie cellulaire et moléculaire du CNRS et du Collège de France, 49bis, Avenue de la Belle-Gabrielle 94736 Nogent-sur-Marne Cédex, France*
*‡School of Human Sciences, Waseda University, 2-579-15 Mikajima, Tokotozawa, Saitama 359, Japan*

In previous experiments, we have demonstrated that limb buds engrafted during embryonic life at E4, between MHC-mismatched chick embryos, are not only tolerated after birth, but induce in the recipient a state of split tolerance toward cells expressing the donor MHC haplotype: donor's skin grafts are permanently tolerated while a proliferative response of host's T cells is generated in MLR by donor–type blood cells. If the same experiment is performed, using quail embryo as a donor and chick as a recipient, acute rejection of the quail limb starts during the first two weeks after birth, thus suggesting that the peripheral type of tolerance induced in these experiments can be obtained only in allogeneic but not in xenogeneic combinations.

We report here the unexpected result that when a chick limb bud is grafted into a quail at E4, it is tolerated and, like allogeneic grafts in chickens, induces adult skin-graft tolerance without modifying the MLR response. Similar results were obtained with grafts from another closely related species of bird, the guinea fowl from the *Phasianidae* family. In contrast, xenogeneic combinations involving more distant species (chick and quail as recipients and duck, an *Anatidae*, as donor) resulted in strong and early rejection from both recipients. As a whole, quails exhibit a greater ability than the chick to become tolerant to antigens presented peripherally from early developmental stages. In adult quails, however, skin grafts performed in either direction (i.e., quail to chick or the reverse) are rejected according to a similar temporal pattern. Moreover, lymphocytes of both species are able to respond equally well to quail or chick IL-2. Several hypotheses are envisaged to account for these observations. It seems likely that this type of tolerance is directly related to antigenic load because the load in chick to quail wing chimeras is larger than that in quail to chick chimeras. This view is supported by the protracted delay in graft rejection observed when two quail wing buds instead of one are grafted into chickens.

KEYWORDS: Xenografts, avian embryo manipulations, peripheral tolerance, quail–chick.

### INTRODUCTION

A series of experiments were initiated in our laboratory a few years ago to test the effect on tolerance induction of embryonic tissue grafts between either two species of birds, the quail (Coturnix coturnix japonica) and the chick (Gallus gallus), both belonging to the same taxonomic family, the *Phasianidae*, or between chickens differing genetically in their major histocompatibility complex (MHC: B locus in birds). The grafts consisted in the replacement of a given embryonic territory of the host embryo by its counterpart from the donor. Transplants were taken prior to vascularization and, therefore, did not include hemopoietic cells (HC) from donor origin, thus making this system fundamentally distinct from that of Billingham et al. (1953) in which tolerance was induced in embryos and neonatal animals by HC transfer. So far, the interspecific grafts have been performed using quail donors and chick hosts and have involved fragments of neuroepithelium (Kinutani and Le Dou-

*Corresponding author.

arin, 1985; Kinutani et al., 1986; Balaban et al., 1988; Kinutani et al., 1989; Hallonet et al., 1990), limb bud (Ohki et al., 1987, 1988), and the epithelio-mesenchymal stroma of the bursa of Fabricius (Belo et al., 1985, 1989; Corbel et al., 1987).

In all cases, the quail organ developed normally in the host embryo with no signs of immune attack from the recipient's developing immune system. When the transplant was strictly restricted to the neuroepithelium, as in truncal neural tube grafts (Kinutani and Le Douarin, 1985; Kinutani et al., 1986, 1989), rejection occurred several weeks to a few months after birth and, in certain strain combinations, even led to reversible damage of the grafted nervous system with complete recovery of motile activity (Kinutani et al., 1989).

In the case of quail to chick limb bud grafts, the beginning of rejection took place within the first 15 days of life, in all cases leading to the complete destruction and autoamputation of the grafted wing (Ohki et al., 1987). For the bursa of Fabricius, in which signs of rejection can be observed only if the animal is sacrificed, the immune attack of the stroma occurs within the first weeks after birth (Corbel et al., 1987).

Within the chicken species, E4-transplantation of limb buds between MHC-mismatched embryos led to virtually complete tissue tolerance not only of the grafted limb, but also of adult skin from the same MHC type as the limb (Corbel et al., 1990). Therefore, it appeared that isochronic grafts of early embryonic organ rudiments performed in the embryo can induce tolerance when done within the same species, but not in quail-to-chick combinations. In the latter case, only thymic epithelial grafts were found to be able to rescue the graft (Ohki et al., 1987, 1988; Belo et al., 1989). It is noteworthy that the immune attack of the host in the case of quail-to-chick grafts is so strong that even neural tissues, which have a special immunological status owing to their lack of class I MHC antigens and to the presence of a blood brain barrier, were subjected, albeit late after birth, to immune destruction by the host. This does not occur in chick-to-chick neural tissue grafts where the chimeras survive normally as control chickens.

The work reported here describes the unexpected observation that as far as limb bud grafts at E4 are concerned, the immune response of the host is very different in chick-to-quail than in quail-to-chick chimeras. We found that embryonic chick wings are well tolerated (with only slight signs of pathology without ultimate rejection) by the quail and as in chick-to-chick MHC mismatched combinations. Moreover, they induce tolerance of MHC-type donor skin grafts in adults.

### RESULTS

### Grafts of a Chick Limb Bud into Quail Embryos

Sixty-four quail embryos received chick-limb bud grafts at E4; 14 of them hatched (22%). These chimeras were designated as ADP (wing of chick in quail host). Two chimeras were sacrificed precociously for histological examination, one (ADP 10) at 3 days posthatching (P3) and the other (ADP 9) at P11.

### Postnatal Evolution of the Graft

Nine chimeras had a normally developed, full-sized wing, whereas in two others (ADP 13, ADP 15), the wing was reduced in size, and in one (ADP 12), it was abnormally shaped. The growth rate and final size of the grafted wing were measured in the chimeras showing a normally shaped graft. Both parameters were larger in the graft than in the contralateral quail wing and reached about the values found in normal chickens (Fig. 1). For example, the length of the ulna was approximately 50 mm at 1 month of age in the grafted wing of ADP 6 as well as in control chick wings, whereas it was about 33 mm in quail wings at the same age.

In contrast with the course of development observed in quail-to-chick limb-bud chimeras, where the grafted wing was strongly rejected within the first weeks posthatching (Ohki et al., 1987), the chick wing grafted into a quail was tolerated with either no rejection or signs of rejection that were mostly limited. For example, in the two chimeras, ADP 1 (Fig. 1c) and ADP 15, which died, respectively, at 155 days (spontaneous death) and 49 days (accidental death), no signs of rejection appeared on the grafted wing.

In the 10 other birds, some signs of rejection appeared at various times after birth (mean: 32.1 ±16 days; Fig. 2), but the immune reaction was

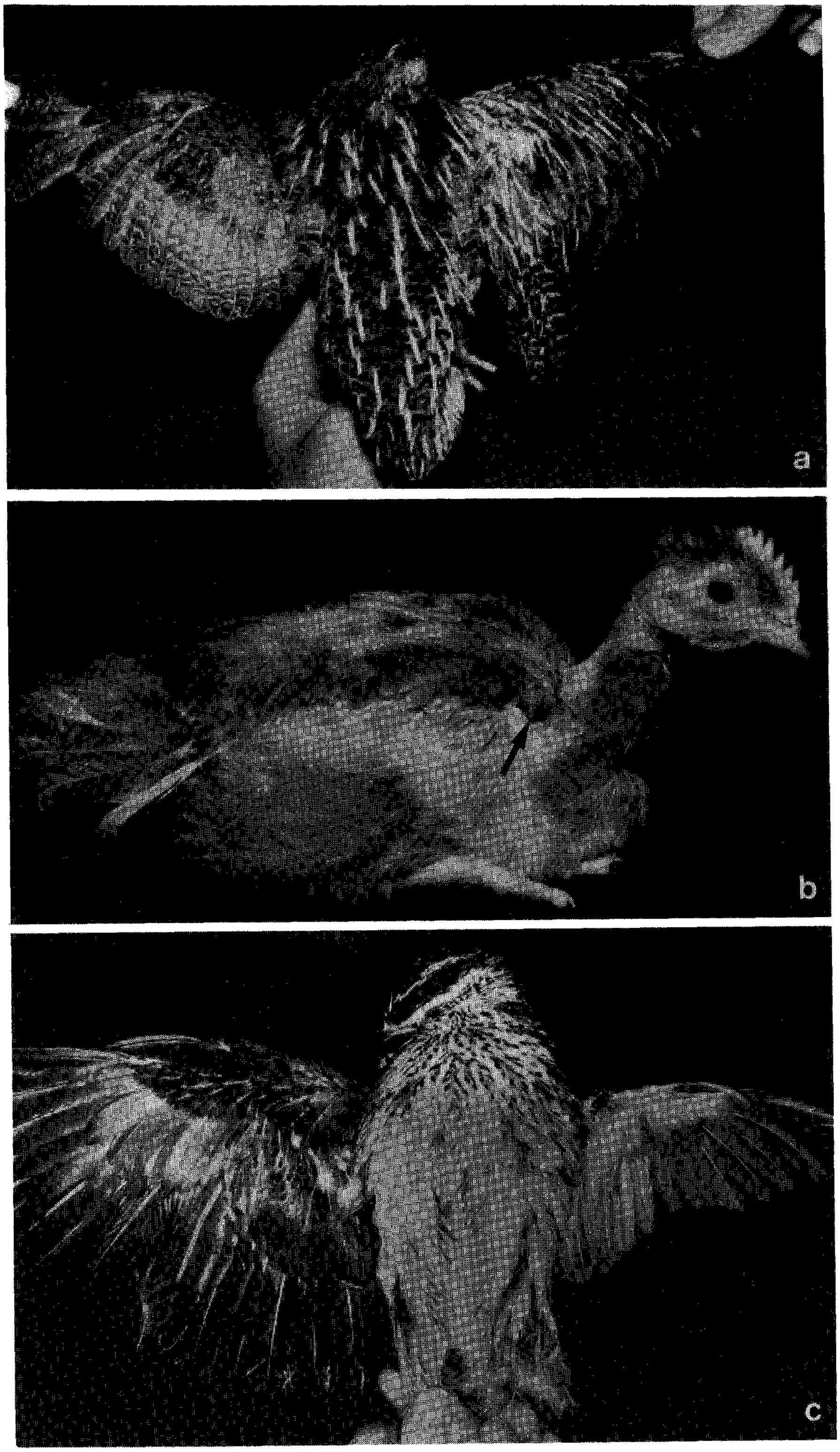

FIGURE 1. Comparative study of the fate of a grafted wing in chick-to-quail and quail-to-chick chimeras. The right limb bud was exchanged between a quail and a chick embryo at E4. (a) ADP6: The quail host (P 30) tolerates the chick wing. (b) ADC510: The chick host of the same age as ADP6 has already rejected the quail wing, which is reduced to a small mass of necrotic tissue (arrow). (c) ADP1: A 3-month-old chick-to-quail chimera. The grafted wing is tolerated.

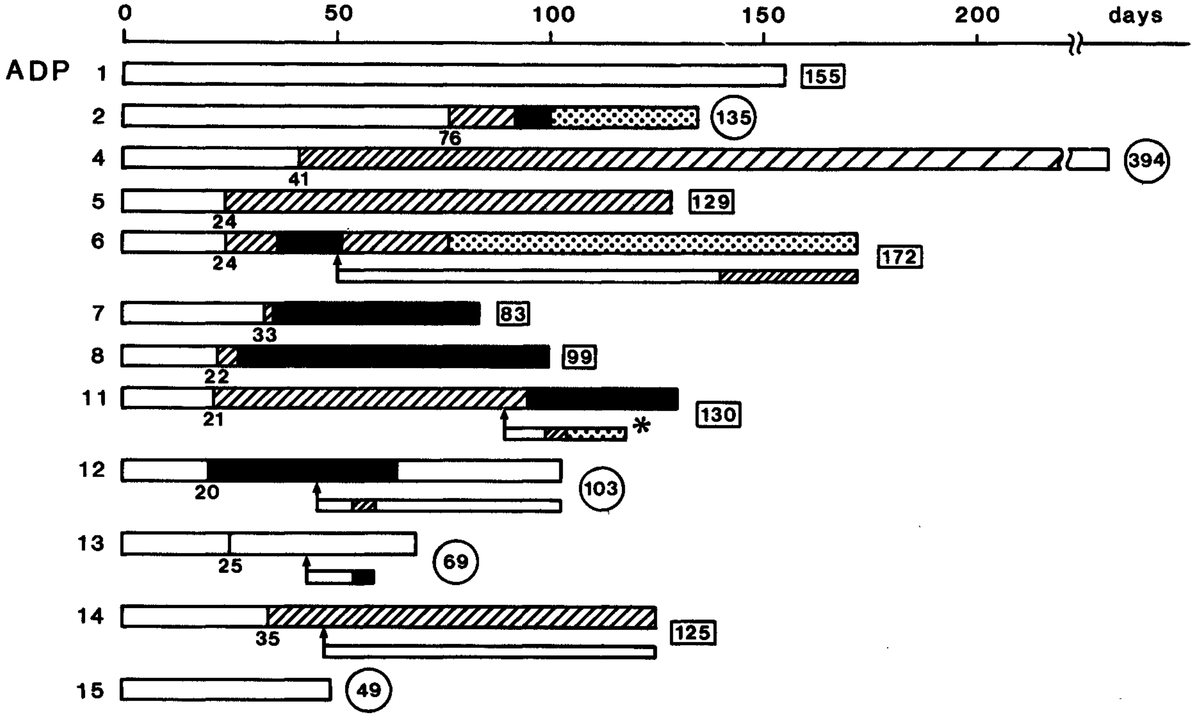

FIGURE 2. Appearance after birth of signs of rejection in the xenogeneic grafted wing and in the adult skin graft of the wing genotype. ↑: age of ADP (days) when the donor-type skin graft was performed. *: the donor-type skin graft was surgically removed and processed for histological examination. □: healthy. ▨: slight rejection. ■: strong rejection. ⓧ : sacrificed at x days. ⊡ : died at y days.

mostly of the chronic type. Three chimeras had moderate and limited inflammation (ADP 4, ADP 5, and ADP 14). In seven chimeras, rejection was stronger with suppuration, formation of blisters and scabs. ADP 7, 8, and 11 died during this period. In contrast, in ADP 2, 6, and 12, the rejection crisis was followed by healing. ADP 11 showed a long chronic alteration of the skin that became worse after 95 days. In all cases, however, the lesions remained essentially superficial, the size of the wing was never reduced, and in no case did the host's immune attack show the dramatic effect observed in the reverse (quail-to-chick) wing grafts. This discrepancy was particularly evident in two experiments in which a reciprocal exchange of wing buds was done between a quail and a chick embryo. This was the case for ADP 4, whose wing was grafted onto chick ADC 509, which provided the wing to the quail recipient. A similar combination was performed between ADP 6 and ADC 510 (Figs. 1a and 1b). Whereas the quail tolerated the chick wing, the quail wing grafted into the chick was precociously rejected.

### Cellular Composition of the Graft at Various Times Posthatching

The species type of the grafted limb tissues was investigated by using the Feulgen–Rossenbeck's staining procedure, which allows quail and chick cells to be distinguished. In ADP 10 sacrificed at P3, most of the tissues were found to be of the donor type as expected. However, Schwann cells lining peripheral nerves were all derived from the quail host. Endothelial cells of blood vessels were mostly of quail type with a small participation (in patches) of chick cells as evidenced with a-MB1 Mab that recognizes a surface glycoprotein of quail but not chick endothelial cells (Péault et al., 1983). This also was expected because the Schwann cells originate from the host's neural crest and the endothelial cells of the limb have been shown to invade the limb-bud rudiment at a stage of development later than the time of the graft (Pardanaud et al., 1989). This shows that, although a few blood capillaries might in some cases have already invaded the

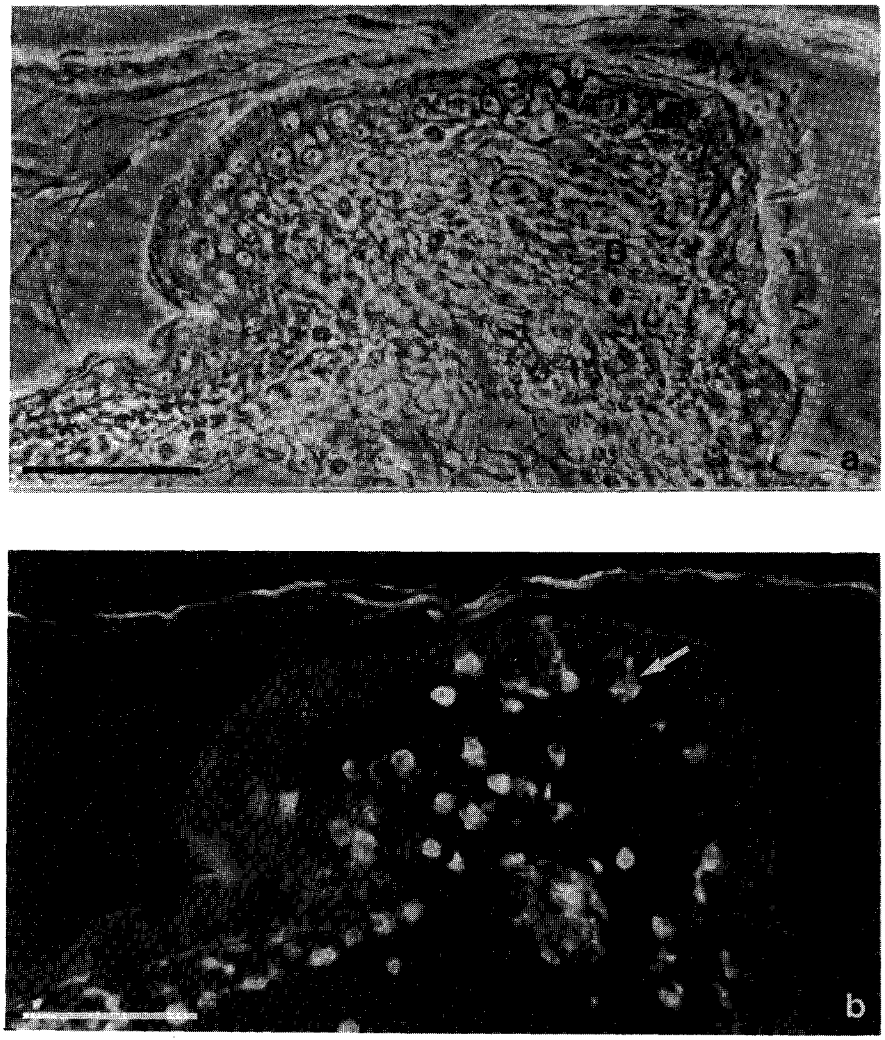

FIGURE 3. Immunostaining with anti-MB1 Mab of skin from the chick-grafted wing and the controlateral quail wing in ADP10, 3 days after hatching. Section of skin from the right chick wing, which has been grafted. (a) Phase contrast view: E, epidermis; D, dermis. (b) MB1-positive hemopoietic cells of the quail can been seen in the dermis. Very few of them are in the epidermis (arrow). MB1 immunoreactivity appears in the epithelial cells, in the hemopoietic and endothelial cells of the dermis. Bar=50 $\mu$m.

limb bud before its removal from the donor, later on the graft is massively colonized by vascular buds from the recipient.

a-MB1 Mab staining also allowed blood-cell infiltration into the donor's limb skin to be visualized. Many cells either round or with elongated processes were interstitially located within the dermis, and even in the epidermis of the graft (Figs. 3a and 3b). They were more numerous than in the equivalent territory of the contralateral quail wing of the recipient.

Examination of ADP 9 sacrificed at P11, while showing slight oedema at the elbow of the grafted wing, revealed basically similar features as ADP 10 except that blood-cell infiltration into the skin of the graft was significantly more abundant (Fig. 4).

### Immunological Response of the Host in MLR and to Skin Grafts

Proliferative responses of the quail peripheral blood T lymphocytes against donor-type stimulator cells were tested in the couple (reciprocal chimeras) formed by ADP 6 and ADC 510. It could be tested also in ADP 11 in which the donor was a (B15xB21) F1. As shown in Table 1, proliferative responses were similar when stimulator cells were of the graft type or from a third

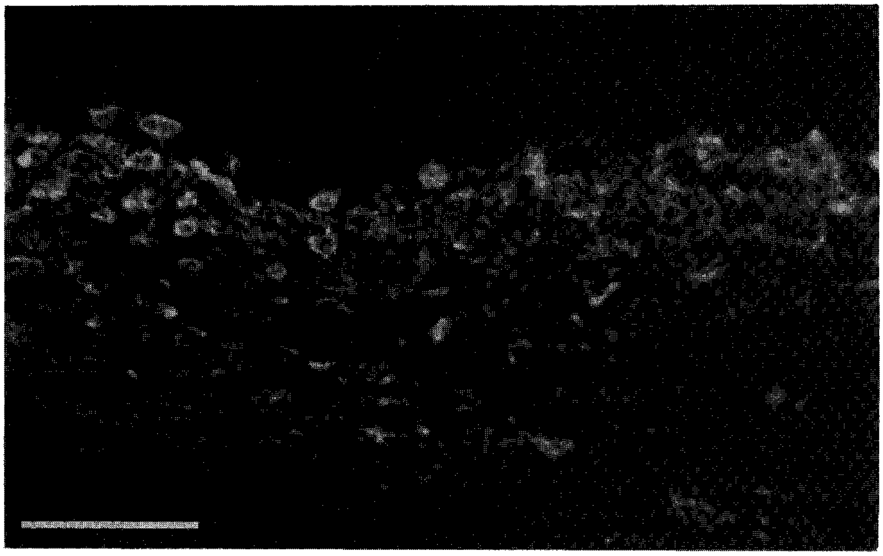

FIGURE 4. Section of skin from the grafted wing in ADP9 labeled with anti-MB1 Mab, at P11. MB1-positive hemopoietic cells are numerous in the entire dermis. Bar=50 $\mu$m.

TABLE 1
MLR Responses of PBL from Two Chick-to-Quail Chimeras (ADP) that Partially Tolerated the Xenogeneic Grafted Wing and Adult Skin Grafts from Donor Type[a]

| Responders | Age[b] | Stimulator Cells | | | |
|---|---|---|---|---|---|
| | | None | Quail | Graft type | Third party |
| ADP6 | P99 | 570±110 | 3140±320 (9.5)[c] | 1670±200[d] (2.9) | 1700±500 (3.0) |
| ADP 11 | P69 | 380±50 | ND[e] | 1940±320 (5.1) | 4080±930 (10.7) |
| | P107 | 360±10 | 720±180 (2.0) | 1080±70 (3.0) | 1330±210 (3.7) |
| Control quail | | 600±450 | 1850±60 (3.1) | 5060±740 (8.4) | 5960±1900 (9.9) |

[a]Proliferation was measured by $^3$H-TdR incorporation after 4 days of culture. Data represent the mean cpm±SD of triplicate culture.
[b]Age of the chimera when the assay was performed (P: posthatching days).
[c]Stimulation index expressed as experimental cpm/cpm from culture without stimulator.
[d]Stimulator cells from ADC 510, wing graft donor, outbred chick transplanted at E 3.5 with a quail limb bud selectively removed from ADP 6 embryonic recipient.
[e]ND: not determined.

party, indicating that, as far as MLR is concerned, no tolerance is induced in the chimeras.

A different result was obtained with skin grafts. As a control, the capacity of adult quails to reject skin grafts from other (outbred) quails and from chickens with different MHC haplotypes was tested. The grafts were promptly rejected, attesting to a normal capacity of the quail species to reject foreign tissues (Table 2).

ADP 6, which received a skin graft from ADC 510 at P50, tolerated the skin perfectly for 3 months. From that time onwards, it exhibited minor chronic signs of rejection both in wattle skin graft and in the chick wing.

ADP 11 showed moderate signs of rejection of the (B15xB21) F1 wing graft when wattle grafts from donor-type chicken were performed. Stronger inflammation of the grafted wing occurred thereafter. At that time, the third-party skin graft had already been rejected. In contrast, the (B15xB21) F1 skin of the wing MHC exhibited only moderate signs of transient inflammation, which later healed. Twenty-eight days after grafting, the (B15xB21) F1 grafted skin was sub-

TABLE 2
Skin Grafts on Adult Control Quail

| Origin of skin | Number of cases | Time of initial rejection mean±SD (days) |
|---|---|---|
| Quail | 6 | 8.8±1.6 |
| Chicken[a] | 17 | 10.4±2.0 |
| Guinea fowl | 5 | 11.3±3.3 |

[a]Skin grafts from different strains of chicken (WL, JA, CC, CB and (WB×M) F1 were performed.

TABLE 3
Restimulation of Quail and Chick ConA-T Blasts Proliferation by Conditioned Media Containing IL-2 from Both Species[a]

| T blasts | Experiment | Addition of CAS from two species | | |
|---|---|---|---|---|
| | | None | Quail | Chick |
| | | Incorporation of $^3$H-TdR (cpm) | | |
| Quail | I | 180±60 | 4270±1340 (23.7) | 3490±630 (19.4) |
| | II | 500±190 | 7050±900 (14.1) | 6490±1060 (13.0) |
| Chick | I | 280±200 | 1130±630 (4.0) | 1650±360 (5.8) |
| | II[b] | 190±25 | 1860±40 (9.8) | 6930±1440 (36.5) |

[a]$2\times10^4$ ConA-T-blasts were cultured with or without 20% conditioned media (CAS) in presence of $\alpha$-methylmannoside (20 mM). $^3$H-thymidine incorporation was measured after 2 days of culture. Stimulation index is given in brackets.
[b]ConA-T-blasts were enriched for CT4-positive cells by FACS.

jected to histological examination. The chick origin of the epidermis, dermis, and blood vessels was confirmed by Feulgen staining: a-MB1 immunofluorescence showed intense quail-blood cell infiltration into the dermis, but the chick tissues were healthy.

Similarly, in ADP 12, a wattle graft of B12 (MHC haplotype of the grafted wing) was performed at P45. After a phase of moderate rejection, the graft appeared perfectly healthy, when the chimera was sacrificed. Histological screening revealed that the graft was made up of chick epidermis, dermis, and contained quail-blood vessels as expected. As in the previously described skin graft, large quail leucocyte infiltrations were also observed.

In all chimeras, the third-party grafts of either quail or chick were rejected as promptly as in normal control quail, showing that when an embryonic graft induces partial or a full tissular tolerance, this tolerance is specific to the tissues carrying the same MHC haplotype than the embryonic graft.

In conclusion, although the proliferative response of the host T lymphocytes against donor antigen expressed by hemopoietic cells can normally be induced *in vitro*, a more or less complete state of tolerance for tissue graft was induced by the embryonic wing graft.

### Cross-Reactivity Between Quail and Chick Cytokines

In an attempt to see whether chick and quail T lymphocytes can be equally well stimulated by isogenically or xenogenically produced IL-2, we devised an *in vitro* assay in which Con A activated PBL supernatant (CAS) from chick was added to quail T blasts and vice versa. In both cases, T-cell proliferation was comparable to that observed when IL-2 of each species was applied to its own T cells. No significant differences were seen in the two experimental series (Table 3), indicating that there is no phylogenetic specificity of CAS produced by the quail and the chick.

### Grafts of Embryonic Wing Bud of Guinea Fowl and Duck into Quail Embryos

We then examined the immune response of both quail and chick species to embryonic grafts from other species of birds. One, the guinea fowl, belonged to the same zoological family as chick and quail, the *Phasianidae*. It was chosen because its incubation time of 27 days is longer than that of the chick. The problem raised was to see if a longer incubation time (it) as it exists between the chick (it: 21 days) and the quail (it: 16 to 17 days) could play a role in the immunological response to embryonic limb bud grafts.

The second species chosen was the duck (*Anatidae*) because it is phylogenetically distant from both chick and quail.

Grafts of limb bud taken from E4 guinea-fowl embryos were performed on quail embryos as described before. Two out of five chimeras hatched and could be examined. As expected, the grafted wing became bigger than its normal quail counterpart. Signs of rejection appeared 2 weeks after hatching with local oedema and some loss of down. Rejection was reversible and the wing did not stop growing and was normally covered by feathers (Fig. 5).

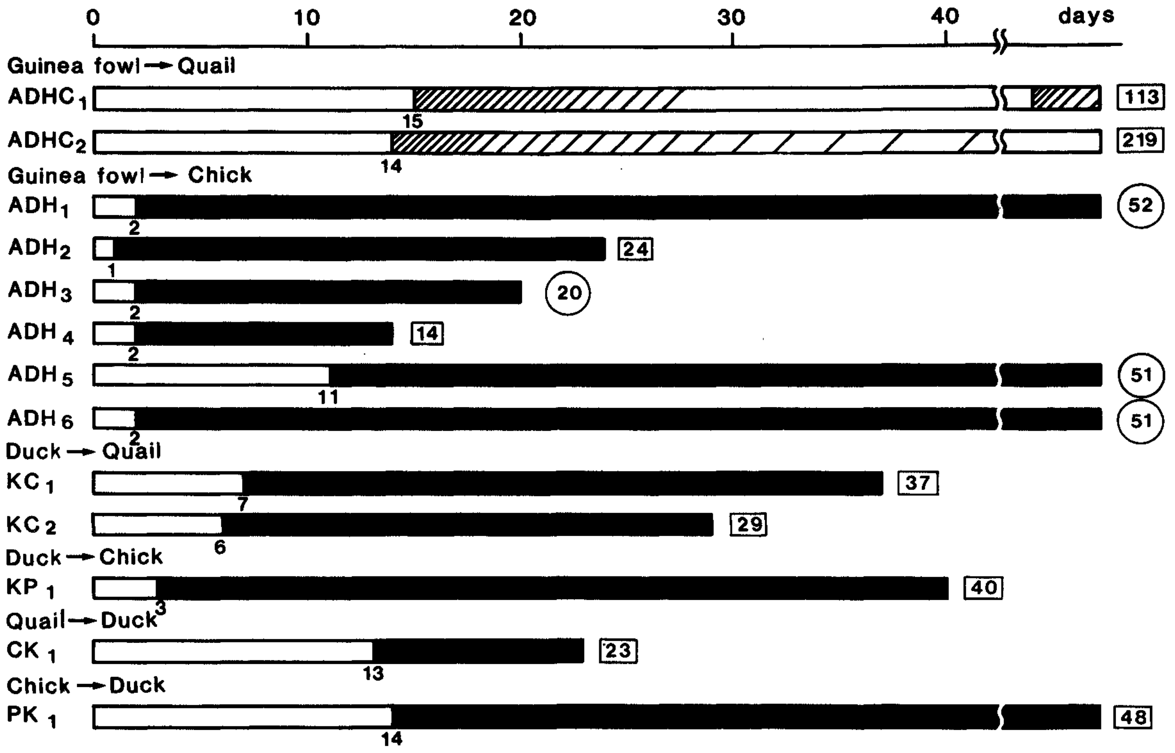

FIGURE 5. Appearance after birth of signs of rejection in the xenogeneic grafted wing. Different species' combinations were performed as indicated. See also the legend of Fig. 2.

Six chimeras (out of 11) in which a limb bud from a guinea fowl was transplanted into a chick embryo survived and hatched. The posthatching fate of the graft was strikingly different to that just described in the guinea fowl-to-quail combination. Rejection occurred as early as P3 and led to the complete destruction of the wing. All the chimeras died during the rejection process.

In duck-to-quail and duck-to-chick combinations, rejection took place from P6–P7 in the former and from P3 in the latter and was in both cases acute. The three birds concerned died precociously during the rejection phase.

E4 quail- and chick-limb buds were also grafted into E5 duck embryos. One bird hatched in each series. In both combinations, acute rejection occurred at P13 and P14, respectively.

### DISCUSSION

This work is part of a larger investigation undertaken with the aim of analyzing the capacity of nonhemopoietic cells to induce tolerance when introduced into an individual during embryogenesis. It was previously found (Corbel et al., 1990) that grafts of limb buds at E4 (i.e., before the onset of thymus ontogeny) between MHC-mismatched chickens yielded wings that not only were tolerated, but also induced tolerance to skin grafts of the same MHC-haplotype. Quail-limb buds introduced into a chick embryo according to a similar experimental design were, however, promptly and acutely rejected after birth. It seemed, therefore, that this sort of tolerance could occur only if graft and donor belonged to the same species and whatever the nature of the mechanisms involved, it could not cross the species barrier. It is actually well known that when induction of tolerance by hemopoietic cell (HC) injection was attempted between two species of birds, although belonging to the same family (chick and turkey), it failed to occur (Hasek, 1956; Mitchison, 1962).

An exception to this rule was brought about when we demonstrated that limb-bud rejection could be permanently prevented in the quail-to-chick limb-bud graft assay by thymic epithelial grafts (Ohki et al., 1987, 1988; Belo et al., 1989).

In this work, we report that in the reverse type of association, that is, chick-to-quail limb-bud grafts, a partial but fairly efficient tolerance of the grafted chick wing is also induced in the absence of thymic graft from the donor of the wing. In fact, the fate of chick-to-quail limb-bud grafts is similar to that of the chick-to-chick combinations just described. The presence of the chick limb during development induces tolerance of the limb-MHC-type skin in the adult chimeras. As with the interspecies grafts, no tolerance was seen in MLR, thus suggesting that the mechanisms involved did not concern clonal deletion of T-cell clones reactive toward the wing antigens in the thymus, but rather in their peripheral inactivation (Corbel et al., 1990).

Other types of species combinations using the limb-bud graft assay into chick or quail embryos involved another *Phasianidae*, the guinea fowl, and an *Anatidae*, the duck (*Anas platyrhynchos*).

The choice of the guinea fowl and duck was driven by two considerations. One was phylogenetic; the other was developmental and related to the duration of incubation of the two protagonists, host and donor. First, the guinea fowl is phylogenetically close to both chicken and quail, whereas the duck is not. Comparison of the results obtained with these two species' wings grafted onto quail and chick was then of interest. It was found that the quail tolerated the guinea fowl wing, albeit after showing slight and recurrent signs of rejection. In contrast, the quail acutely and promptly rejected the duck graft. In this case, one can invoke the phylogenetic distance to explain the difference in the reaction of the quail immune system to the antigens carried by these two species.

The response of the chick to a duck-grafted wing was the same as that of the quail, and in the reverse association, that is, for duck embryos grafted with quail or chick wing, no tolerance was observed. This reinforces the interpretation according to which tolerance by early exposure of foreign antigens to the developing immune system can occur only between closely related species.

The developmental consideration that justified the choice of the guinea fowl deals with its incubation time of 27 days. One possible reason for the difference of reaction of quail and chicken in the reverse grafts could possibly be related to the difference in the incubation period, which is 17 days for the quail and 21 days for the chick. Differences in the maturation of the immune system and in the expression of MHC cell type and of species-specific antigens in graft and host could play a role in the capacity of a given species to induce tolerance in the other. Combination of chick-host and guinea-fowl grafts then reproduces the situation of quail host and chicken grafts as far as this parameter is concerned. If it plays a role at all in the phenomenon observed, it should either delay or prevent rejection of the guinea fowl wing by the chick host. This did not occur because rejection was precocious and acute in this case as it was for the quail wing.

It appears from these experiments that the quail immune system consistently has a capacity to tolerate tissues of relatively closely related species, a capacity that does not exist in the chick. We have not yet determined if it is the chick or the quail that exhibits the less common type of behavior in this respect.

In the absence of any experimental data, one can speculate that the reason for which the quail behaves with regard to xenogeneic grafts (from *Phasianideae*) as the chick does to allogeneic transplants, relies on differences in the germ-line repertoire of T-cell receptor (TCR) genes in the two species.

If the quail TCR repertoire is wider than that of the chick, it may be able to recognize chick antigenic determinants, that is, chick MHC (alleles+peptides), as it does for the variety of antigens of its own species (alloantigens). The chick would have a TCR repertoire strictly adjusted to the MHC antigens of its own species. In this respect, the chick would fit with Jerne's hypothesis (1971) that the germ-line repertoires of a given species are essentially selected for the recognition of the MHC (or MHC+peptides) of this particular species. With a more limited repertoire, it would then be unable to adjust the selection mechanisms involved in this particular type of tolerance induction to quail or guinea fowl (closely related to itself in taxonomy though they are). In any case, a barrier exists between chickens and other species for this type of peripheral mechanism of tolerance induction that can be overcome only by thymus epithelial grafts (Le Douarin et al., 1990), a phenomenon that has recently been considered as triggered by a mechanism of clonal anergy rather than clonal deletion (Ramsdell et al., 1989).

It is important to notice that adult quails reject skin grafts of either allogeneic or xenogeneic origin as promptly as the other species of birds so far investigated, and namely as the chick. Therefore, one cannot invoke a generally low immune rejection capacity of the quail to explain the phenomena reported here.

In the numerous reports found in the literature, xenogeneic immunological responses show an extreme variability according to both the species combinations and the assay considered. For example, some authors stated that xenogeneic MLR stimulation (Wilson and Nowell, 1970) as well as graft-versus-host responses are weaker than allogeneic (review in Simonsen, 1962, and references therein; Lafferty and Jones, 1969). In contrast, Widmer and Bach (1972) found that the MLR responses can be as important in xenogeneic as in allogeneic combinations and that there is a great variation in the extent to which xenogeneic associations may respond. Recently, Alter and Bach (1990) confirmed that the human-cell repertoire does include the ability to recognize very widely disparate (i.e., murine) xenoantigens.

With the aim of determining if some sort of species incompatibility could be detected at the functional level between quail and chick immune systems, we investigated the reciprocal effect of quail and chick IL-2 on T cells from the other species. Indeed, an absence of reciprocity in the response to IL-2 has been already observed between certain mammalian species (Robb et al., 1981; Alter and Bach, 1990). We found here that between quail and chick, IL-2 of either origin promotes proliferation of T cells from both species.

In line with the concept proposed by Lafferty et al. (1983), another reason for the lack of reactivity toward the graft in the chick-to-quail combination could be the absence in the chimera of cells able to present the foreign antigens to the host's T cells and/or to deliver appropriate "second signals" that might be either cell-bound or soluble lymphokines. This is an unlikely possibility since such a problem does not hold in the reverse graft (quail to chick), where the developmental stages of host and donor are the same. Moreover, it is noteworthy that grafting adult skin from the chick donor strain, carrying donor hemopoietic cells, not only did not alter the privileged immunological status of the grafted limb, but were themselves tolerated. Therefore, in this aspect of the immune response, no evident discrepancy could be found between the quail and the chick species.

A possible cause that could be put forward to account for the different response of the host toward embryonic limb-bud grafts is the size of the graft, that is, the antigenic load. In the suppressive type of effect observed in these experiments, one could expect that the larger the graft becomes in the adult, the stronger the effect should be. This is not an unlikely possibility since we actually found that increasing the antigenic load in the quail-to-chick limb-bud graft assay (i.e., grafting two quail limb buds instead of one) consistently delayed the onset of the immune response. When one quail wing was implanted into a chick at E4, graft rejection always started before P15 and was readily and consistently acute (Ohki et al., 1987). In contrast, 36% of the animals engrafted with two quail wings, in similar circumstances, started to reject the graft between P15 and P25, and the rejection was in several cases of the chronic type rather than acute (our unpublished observations).

It is also remarkable to notice that tolerance of the chick wing by the quail involves extensive infiltration of leucocytes from the host, thus suggesting that a certain amount of activation of the host's immune system takes place. The fundamentally healthy state of the graft seems, therefore, to be the result of an equilibrium between two antagonistic tendencies: activation of competent immune cells and their suppression by an unknown mechanism. This equilibrium presents a certain level of instability attested to by the transient rejection crises emerging from time to time during the lifespan of the recipient. This observation is relevant to the view presented by Coutinho and Bandeira (1989) that, in addition to the passive mechanism based upon clonal deletion, tolerance actually involves active regulatory processes. These authors thus interpret the fact that, in the classical system of transplantation, where tolerance is induced by neonatal injection of semiallogeneic hemopoietic cells, high levels of T and B cell activity are always observed (Bandeira et al., 1989).

Another case of tolerance induction by grafts of nonthymic, nonhemopoietic cells in embryos has been described. It concerns implantation into frog embryos of allogeneic eye anlagen, resulting in long-term survival of skin grafts of the eye donor haplotype in adults (Flajnik et al., 1985). Moreover, the transgenic mice system has been extremely informative in the last years in showing that an alternative type of tolerance, based on clonal nonresponsiveness rather than clonal deletion, operates extrathymically (Lo et al., 1988; Sarvetnick et al., 1988; Böhme et al., 1989; Morahan et al., 1989; Murphy et al., 1989; Miller et al., 1990).

We demonstrate here that such mechanisms can be operative between different species provided that they are closely related in taxonomy. Moreover, our experiments suggest that the antigenic load, along with the early exposure of the antigens to the immune system, is critical for the equilibrium necessary for tolerance to be effective.

### MATERIALS AND METHODS

Fertilized eggs of outbred ducks (*Anas platyrhynchos*), quail (*Coturnix coturnix japonica*), guinea fowls (*Numida meleagris*), and of chickens (*Gallus gallus*) of white Leghorn and JA strains from commercial sources were used. We also used embryos from the inbred lines CC (B4-MHC haplotype), CB (B12-MHC haplotype), and F1 from a cross between the two inbred lines WB and M (MHC: B15×B21). These lines were established in Czechoslovakia (Hala et al., 1966; Hasek et al., 1966) and maintained by Dr. Hlozanek (Institute of Molecular Genetics, Praha), who provided us with eggs.

Embryonic transplantation of the wing bud was performed as previously described (Ohki et al., 1987). The right forelimb bud of the donor embryo was substituted for its counterpart in a recipient. Both host and donor embryos were at the same developmental stage, 3.5 to 4 days of incubation (E3.5–E4), that is, stages 18 to 23 according to Hamburger and Hamilton (1951) for the chick and stages 15 to 17 according to Zacchei (1961) for the quail. Platinum staples maintained the graft and allowed rapid vascularization of the grafted wing rudiment.

### Adult Skin Grafts

A piece of chick wattle of about 1 $cm^2$ was grafted and sutured on the back of a quail under pentobarbital anesthesia. The skin graft was inspected daily.

### Histological Procedures

At autopsy, the grafted wattle and pieces of skin and muscles taken from the grafted wing were processed for histological examination after fixation in Zenker's fluid and stained according to the Feulgen–Rossenbeck's technique, which allows quail and chick cells to be distinguished by the structure of their interphase nuclei (Le Douarin, 1969, 1973).

Immunofluorescence staining of tissue sections fixed in Bouin's solution was performed with a monoclonal antibody (Mab) prepared in our laboratory: anti-MB1 (Péault et al., 1983). The MB1 antigen is expressed by hemopoietic and endothelial cells of the quail and not by their chicken counterparts (Péault et al., 1983; Labastie et al., 1986). Anti-MB1 also stains the quail epidermis after birth (unpublished data).

### Mixed Lymphocyte Reaction

Peripheral blood leucocytes (PBL) were obtained from the supernatant of heparinized blood centrifuged at low speed (60×G) for 20 min at room temperature. Both the responder and stimulator cells were obtained from PBL. Stimulator cells were growth-inhibited by mitomycin C. Mixed leucocyte cultures were done as previously described (Corbel et al., 1990).

Cultures containing equal numbers of responder and stimulator cells ($1\times10^6$) were pulsed, during the last 18 hr, with 1 $\mu$Ci [$^3$H]-thymidine (=37 kbq, specific activity: 25 Ci/mM, Amersham, UK). Results are expressed as cpm (counts per minute) per culture.

### Interleukin-2 Assay

IL-2 containing conditioned media (CAS, Con A activated blood-cell supernatant) was obtained from chick and quail PBL ($3\times10^6$/ml) stimulated with 10 $\mu$g/ml concanavalin A (Con A; Pharmacia Fine Chemicals, Sweden) in Iscove's modified Dulbecco's Medium (IMDM), supplemented with

5% selected fetal calf serum (FCS) and $5\times10^{-5}$ M 2-mercaptoethanol, for 48 hr at 40°C, 5% $CO_2$, as described previously (Corbel and Thomas, 1990).

IL-2 activity in quail-CAS and chick-CAS was assayed by proliferation of Con A-activated T blasts from both species. T blasts activated for 48 hr with Con A were separated from nonactivated and dead cells by centrifugation on a layer of FCS.

After extensive washing in HBSS, they were cultured at a density of $1\times10^5$ cells/ml in 0.2 ml IMDM containing 5% FCS with 20 mM methylmannoside (Sigma Chem, St. Louis), a specific Con A inhibitor, for 2 days in the presence of 20% of CAS.

In one experiment, CD4-positive T blasts were obtained by FACS after indirect immunostaining with anti-CD4 Mab (Chan et al., 1988).

Proliferation was measured by incorporation of 1 $\mu$Ci $^3$H-thymidine per culture for 2 hr.

### ACKNOWLEDGMENTS

We thank Drs. A. Coutinho and C. Ordahl for critically reading the manuscript; K. Heydon for technical assistance; S. Gournet, Y. Rantier, and B. Henri for contributions to the illustrations; and E. Bourson for the preparation of the typescript.

This work was supported by the Centre National de la Recherche Scientifique, the Institut National de la Santé et de la Recherche Médicale and by grants from the following Foundations: the Association pour la Recherche Médicale and the Ligue Nationale Française contre le Cancer.

*(Received March 4, 1991)*

*(Accepted March 12, 1991)*

### REFERENCES

Alter B.J., and Bach F.H. (1990). Cellular basis of the proliferative response of human T cells to mouse xenoantigens. J. Exp. Med. **171:** 333–338.

Balaban E., Teillet M.A., and Le Douarin N.M. (1988). Application of the quail–chick chimera system to the study of brain development and behavior. Science **241:** 1339–1342.

Bandeira A., Coutinho A., Carnaud C., Jacquemart F., and Forni L. (1989). Transplantation tolerance correlates with high levels of T- and B-lymphocyte activity. Proc. Natl. Acad. Sci. USA **86:** 272–276.

Belo M., Corbel C., Martin C., and Le Douarin N.M. (1989). Thymic epithelium tolerizes chickens to embryonic grafts of quail bursa of Fabricius. Int. Immunol. **1:** 105–112.

Belo M., Martin C., Corbel C., and Le Douarin N.M. (1985). A novel method to bursectomize avian embryos and obtain quail–chick bursal chimaeras. I. Immunocytochemical analysis of such chimaeras by using species-specific monoclonal antibodies. J. Immunol. **135:** 3785–3794.

Billingham R.E., Brent L., and Medawar P.B. (1953). Actively acquired tolerance of foreign cells. Nature **172:** 603–606.

Böhme J., Haskins K., Stecha P., van Ewijk W., LeMeur M., Gerlinger P., Benoist C., and Mathis, D. (1989). Transgenic mice with I-A on islet cells are normoglycemic but immunologically intolerant. Science **244:** 1179–1183.

Chan M.M., Chen C.L.H., Lanier-Ager L., and Cooper M.D. (1988). Identification of the avian homologues of mammalian CD4 and CD8 antigens. J. Immunol. **140:** 2133-2138.

Corbel C., Belo M., Martin C., and Le Douarin N.M. (1987). A novel method to bursectomize avian embryos and obtain quail–chick bursal chimeras. II. Immune response of bursectomized chicks and chimeras and postnatal rejection of the grafted quail bursas. J. Immunol. **138:** 2813–2821.

Corbel C., Martin C., Ohki H., Coltey M., Hlozanek I., and Le Douarin N.M. (1990). Evidence for peripheral mechanisms inducing tissue tolerance during ontogeny. Int. Immunol. **2:** 33–40.

Corbel C., and Thomas J-L. (1990). Establishment of an IL-2 dependent, antigen nonspecific chicken T-cell line. Dev. Comp. Immunol. **14:** 439–446.

Coutinho A., and Bandeira A. (1989). Tolerize one, tolerize them all: Tolerance is self-assertion. Immunol. Today **10:** 264–266.

Flajnik M.F., Du Pasquier L., and Cohen N. (1985). Immune responses of thymus/lymphocyte embryonic chimeras: studies on tolerance and major histocompatibility complex restriction in Xenopus. Eur. J. Immunol. **15:** 540–547.

Hala K., Hasek M., Hlozanek I., Hort J., Knizetova F., and Mervartova H. (1966). Syngeneic lines of chickens. II. Inbreeding and selection within the M, W, and I lines and crosses between the C, M, and W lines. Folia Biol. **12:** 407–422.

Hallonet M.R., Teillet M.A., and Le Douarin N.M. (1990). A new approach to the development of the cerebellum provided by the quail chick marker system.. Development **108:** 19–31.

Hamburger V., and Hamilton H.L. (1951). A series of normal stages in the development of the chick embryo. J. Morph. **88:** 49–92.

Hasek M. (1956). Tolerance phenomena in birds. Proc. Roy. Soc. B **146:** 67–77.

Hasek M., Knizetova F., and Mervartova H. (1966). Syngeneic lines of chickens. I. Inbreeding and selection by means of skin grafts and tests for erythrocyte antigens in C line chickens. Folia Biol. **12:** 335–341.

Jerne N.K. (1971). The somatic generation of immune recognition. Eur. J. Immunol. **1:** 1–9.

Kinutani M., Coltey M., and Le Douarin N.M. (1986). Postnatal development of a demyelinating disease in avian spinal cord chimeras. Cell **45:** 307–314.

Kinutani M., and Le Douarin N.M. (1985). Avian spinal cord chimeras: I. Hatching ability and posthatching survival in homo- and heterospecific chimeras. Dev. Biol. **111:** 243–255.

Kinutani M., Tan K., Desaki J., Coltey M., Kitaoka K., Nagano Y., Takashima Y., and Le Douarin N.M. (1989). Avian spinal cord chimeras. Further studies on the neurological syndrome affecting the chimeras after birth. Cell Diff. and Dev. **26:** 145–162.

Labastie M.C., Poole T.J., Péault B.M., and Le Douarin N.M. (1986). MB1, a quail leukocyte–endothelium antigen: Partial characterization of the cell surface and secreted forms in cultured endothelial cells. Proc. Natl. Acad. Sci. USA **83:** 9016–9020.

Lafferty K.J., and Jones M.A.S. (1969). Reactions of the graft versus host (GvH) type. Aust. J. Exp. Biol. Med. Sci. **47:** 17–25.

Lafferty K.J., Prowse S.J., and Simeonovic C.J. (1983). Immunobiology of tissue transplantation: A return to the passenger leukocyte concept. Ann. Rev. Immunol. **1:** 143–173.

Le Douarin N.M. (1969). Particularités du noyau interphasique chez la Caille japonaise (Coturnix coturnix japonica). Utilisation de ses particularités comme "marquage biologique" dans des recherches. Bull. Biol. Fr. Belg. **103,** 435–452.

Le Douarin N.M. (1973). A biological cell labeling technique and its use in experimental embryology. Dev. Biol. **30:** 217–222.

Le Douarin N.M., Corbel C., Martin C., Coltey M., and Salaün J. (1990). Induction of tolerance by embryonic thymic epithelial grafts in birds and mammals. Cold Spring Harbor Symp. Quant. Biol. **54:** 777-787.

Lo D., Burkly L.C., Widera G., Cowing C., Flavell R.A., Palmiter R.D., and Brinster R.L. (1988). Diabetes and tolerance in transgenic mice expressing class II MHC molecules in pancreatic beta cells. Cell **53:** 159–168.

Miller J., Daitch L., Rath S., and Selsing E. (1990). Tissue specific expression of allogeneic class II MHC molecules induces neither tissue rejection nor clonal inactivation of alloreactive T cells. J. Immunol. **144:** 334–341.

Mitchison N.A. (1962). Tolerance of erythrocytes in poultry: induction and specificity. Immunol. **5:** 341–358.

Morahan G., Allison J., and Miller J.F.A.P. (1989). Tolerance of class I histocompatibility antigens expressed extrathymically. Nature **339:** 622–624.

Murphy K.M., Weaver C.T., Elish M., Allen P.M., and Loh D.Y. (1989). Peripheral tolerance to allogeneic class II histocompatibility antigens expressed in transgenic mice: Evidence against a clonal deletion mechanism. Proc. Natl. Acad. Sci. USA **86:** 10034–10038.

Ohki H., Martin C., Corbel C., Coltey M., and Le Douarin N.M. (1987). Tolerance induced by thymic epithelial grafts in birds. Science **237:** 1032–1035.

Ohki H., Martin C., Coltey M., and Le Douarin N.M. (1988). Implants of quail thymic epithelium generate permanent tolerance in embryonically constructed quail/chick chimeras. Development **104:** 619–630.

Pardanaud L., Yassine F., and Dieterlen-Lièvre F. (1989). Relationship between vasculogenesis, angiogenesis and haemopoiesis during avian ontogeny. Development **105:** 473–485.

Péault B.M., Thiery J.P., and Le Douarin N.M. (1983). Surface marker for the hemopoetic and endothelial cell lineages in quail that is defined by a monoclonal antibody. Proc. Natl. Acad. Sci. USA **80:** 2976–2980.

Ramsdell, F., Lantz, T., and Fowlkes, B.J. (1989). A nondeletional mechanism of thymic self tolerance. Science. **246:** 1038–1041.

Robb R.J., Munck A., and Smith K.A. (1981). T cell growth factor receptors. Quantification, specificity, and biological relevance. J. Exp. Med. **154:** 1455–1474.

Sarvetnick N., Liggitt D., Pitts S.L., Hansen S.E., and Stewart T.A. (1988). Insulin-dependent diabetes mellitus induced in transgenic mice by ectopic expression of class II MHC and interferon-gamma. Cell **52:** 773–782.

Simonsen M. (1962). Graft versus host reactions: Their natural history and applicability as tools of research. Prog. Allergy **6:** 349–467.

Widmer M.B., and Bach F.H. (1972). Allogeneic and xenogeneic response in mixed leukocyte cultures. J. Exp. Med. **135:** 1204–1208.

Wilson D.B., and Nowell P.C. (1970). Quantitative studies on the mixed lymphocyte interaction in rats. IV. Immunological potentiality of the responding cells. J. Exp. Med. **131:** 391–399.

Zacchei A.M. (1961). Lo sviluppo embrionale della quaglia giapponese (Coturnix coturnix japonica T.e S.) Arch. Ital. Anat. Embryonal. **66:** 36–62.

